# To the Editors of the Medico-Chirurgical Journal & Review

**Published:** 1817-11

**Authors:** W. D. Rolfe

**Affiliations:** Surgeon Accoucheur. Bristol


*To the Editors of the Medico-Chirurgical Journal & Review.*

GENTLEMEN,

WISHING to add a little to your useful pages, I beg you will insert the following Case:

A woman, by the name of Keene (widow of the late Sergeant Keene, whose death was so respectfully recorded in the news-papers a few months ago), was admitted into the Bristol Dispensary as a lying-in patient: she supposed herself to be then between seven and eight months with child. She had been subject to slight discharges of blood for some time, which she attributed to the fright experienced on hearing of the death of her husband; but this day the discharge was so profuse, that she was attended by the resident man-midwife, who, upon examination, conceived the case to be attachment of the placenta over the os uteri. He then sent for me, being one of the men-midwives extraordinary to the charity. I found the patient had lost a great deal of blood; her countenance much sunk, with a very low pulse; I accordingly advised immediate delivery. Finding, as might naturally be expected from the condition of the patient, a favourable state of the os uteri; my hand was introduced with ease into the uterus, but, to my great surprise, there was no child; and, upon moving my hand a little, I found the uterus filled with a softish substance that was very easily detached from it. A portion of this protruded through the os uteri, and in its confined situation gave the feel of a placenta. I detached the whole of it from the uterus, and brought it away. When examined, it was found to be a vast quantity of hydatids, from the size of a small pea to that of a pullet's egg: the whole mass weighed more than four pounds. The uterus readily contracted, and the woman has had a perfect recovery.

I am, Gentlemen,
Your obedient servant,
W. D. ROLFE,
Surgeon Accoucheur.

*Bristol,* 11*th Aug.* 1817.

